# Supplementary material for: Long-Term Effects of Air Pollutants on Mortality Risk in Patients with End-Stage Renal Disease
Source: Int J Environ Res Public Health. 2020 Jan 15;17(2):546. doi: 10.3390/ijerph17020546 (PMC7014206; doi:10.3390/ijerph17020546)
Supplement: Supplementary file 1 [file ijerph-17-00546-s001.pdf]

Supplemental Materials for

Long-term effects of air pollutants on mortality risk in patients with end-stage renal disease

Jiyun Jung[1¶], Jae Yoon Park[2,3¶], Yong Chul Kim[4], Hyewon Lee[5,6], Ejin Kim[1], Yong-Lim Kim[7], Yon Su Kim[4], Jung Pyo Lee[4,8]*, Ho Kim[1]*;

Clinical Research Center for End Stage Renal Disease (CRC for ESRD) investigators

*corresponding authors. Email: nephrolee@gmail.com and hokim@snu ac.kr

This file includes:

- Table S1. Concentration of air pollutants from 1 year to 7 years ago on enrollment date of cohort participants
- Table S2. Alkaike information criterion (AIC) of air pollutants by exposure years from 1-year to 7-year in time-varying model
- Table S3. Hazard ratio and 95% confidence interval of ESRD patients when exposed to air pollutants in baseline Cox model.
- Figure S1. Concentration-mortality association (blue line) and 95% confidence interval (grey shadow) adjusted by natural spline with 2 degree of freedom in time-varying Cox model, with the percent distribution of population (histogram)
- Figure S2. Correlation between daily concentrations of air pollutants between 2001 and 2015
- Figure S3. The association between mortality of ESRD patients and exposure to air pollutants in single- and two-pollutant models using time-varying (1yr-7yr) model

Table S1. Concentration of air pollutants from 1 year to 7 years ago on enrollment date of cohort participants

	Exposure years						
	1yr	2yr	3yr	4yr	5yr	6yr	7yr
PM_{10} (μg/m^3)							
IQR	7.54	6.65	7.6	6.91	6.92	7.23	8.14
Mean ± SD	50.38 ± 5.29	51.32 ± 4.95	52.33 ± 4.94	53.32 ± 4.96	54.05 ± 4.97	54.61 ± 5.02	55.22 ± 5.28
Min	33.81	35.69	38.43	40.01	41.01	40.83	41.71
25th	46.63	47.99	48.89	50.32	50.5	50.92	51.26
50th	50.54	51.33	52.19	53.04	53.51	54.15	55.11
75th	54.18	54.64	56.49	57.22	57.42	58.15	59.41
Max	62.72	61.45	62.52	64.65	64.42	65.03	65.52
NO_2 (ppb)							
IQR	11.62	10.91	11.82	12.54	12.94	12.81	12.77
Mean ± SD	27.09 ± 6.66	27.5 ± 6.86	27.77 ± 6.98	27.85 ± 6.98	27.87 ± 6.93	28.03 ± 6.83	28.13 ± 6.89
Min	8.31	9.12	9.44	9.66	9.74	10.05	10.41
25th	21.51	22.50	22.72	22.83	22.88	22.87	22.86
50th	28.92	29.13	29.38	29.55	29.44	29.11	28.92
75th	33.13	33.41	34.54	35.37	35.82	35.68	35.63
Max	36.90	37.61	37.48	37.02	36.62	36.05	36.78
SO_2 (ppb)							
IQR	0.50	0.68	0.81	0.67	0.53	0.39	0.38
Mean ± SD	5.21 ± 0.97	5.37 ± 0.98	5.5 ± 0.98	5.56 ± 0.94	5.6 ± 0.9	5.6 ± 0.88	5.6 ± 0.88
Min	2.09	2.20	2.33	2.44	2.49	2.51	2.62
25th	4.93	5.02	5.11	5.21	5.38	5.51	5.49

50th	5.27	5.33	5.52	5.68	5.70	5.60	5.52
75th	5.43	5.70	5.92	5.88	5.91	5.90	5.87
Max	8.59	8.10	8.14	8.03	7.98	8.20	8.33

Table S2. Alkaike information criterion (AIC) of air pollutants by exposure years from 1-year to 7-year in time-varying model

	Exposure years						
	1yr	2yr	3yr	4yr	5yr	6yr	7yr
PM_{10} (μg/m^3)	18180	18180	18179	18175	18175	18175	18172
NO_2 (ppb)	18177	18178	18178	18177	18177	18176	18176
SO_2 (ppb)	18169	18171	18171	18173	18173	18174	18174

Table S3. Hazard ratio and 95% confidence interval of ESRD patients when exposed to air pollutants in baseline Cox model

	Exposure years						
	1yr	2yr	3yr	4yr	5yr	6yr	7yr
PM_{10} (μg/m^3)							
IQR increment	1.20 (1.06, 1.36)	1.17 (1.05, 1.31)	1.22 (1.06, 1.41)	1.21 (1.05, 1.40)	1.25 (1.08, 1.46)	1.26 (1.07, 1.47)	1.24 (1.06, 1.45)
Q2	1.26 (1.02, 1.55)	1.44 (1.16, 1.78)	1.57 (1.26, 1.96)	1.40 (1.13, 1.74)	1.11 (0.91, 1.35)	1.08 (0.89, 1.30)	1.09 (0.89, 1.32)
Q3	1.26 (1.01, 1.56)	1.09 (0.88, 1.35)	1.26 (0.99, 1.60)	1.20 (0.94, 1.54)	1.03 (0.77, 1.37)	1.06 (0.79, 1.43)	0.97 (0.76, 1.23)
Q4	1.58 (1.23, 2.03)	1.61 (1.25, 2.08)	1.62 (1.24, 2.14)	1.49 (1.13, 1.96)	1.48 (1.12, 1.95)	1.41 (1.06, 1.86)	1.47 (1.09, 1.97)
Trend p-value	<0.01	<0.01	<0.01	0.01	<0.01	<0.01	0.02
NO_2 (ppb)							
IQR increment	1.30 (1.02, 1.66)	1.24 (0.98, 1.57)	1.28 (0.99, 1.65)	1.31 (0.99, 1.75)	1.34 (0.99, 1.82)	1.28 (0.96, 1.70)	1.32 (1.00, 1.75)
Q2	0.99 (0.84, 1.17)	1.08 (0.91, 1.28)	1.07 (0.90, 1.26)	1.05 (0.89, 1.25)	1.00 (0.85, 1.19)	1.04 (0.88, 1.23)	1.05 (0.89, 1.25)
Q3	0.54 (0.35, 0.83)	0.46 (0.29, 0.73)	0.53 (0.34, 0.84)	0.48 (0.30, 0.77)	0.41 (0.23, 0.71)	0.40 (0.22, 0.71)	0.39 (0.21, 0.70)
Q4	0.47 (0.28, 0.78)	0.38 (0.22, 0.65)	0.43 (0.25, 0.74)	0.40 (0.23, 0.69)	0.34 (0.19, 0.62)	0.35 (0.19, 0.66)	0.33 (0.18, 0.63)
Trend p-value	0.03	0.05	0.06	0.03	0.02	0.06	0.15
SO_2 (ppb)							
IQR increment	1.06 (1.03, 1.10)	1.08 (1.03, 1.13)	1.08 (1.02, 1.14)	1.06 (1.01, 1.11)	1.04 (1.00, 1.08)	1.03 (1.00, 1.06)	1.03 (1.00, 1.06)
Q2	1.32 (1.09, 1.61)	0.95 (0.78, 1.16)	1.19 (0.96, 1.48)	1.20 (0.96, 1.49)	1.22 (0.97, 1.53)	1.35 (1.05, 1.75)	1.11 (0.85, 1.46)
Q3	1.39 (1.15, 1.68)	1.26 (1.04, 1.51)	1.23 (1.01, 1.50)	1.06 (0.86, 1.31)	1.21 (0.95, 1.54)	1.19 (0.97, 1.46)	1.24 (1.04, 1.49)
Q4	1.56 (1.28, 1.92)	1.27 (1.02, 1.57)	1.22 (0.98, 1.53)	1.37 (1.09, 1.73)	1.19 (0.97, 1.47)	1.17 (0.95, 1.44)	1.14 (0.92, 1.40)
Trend p-value	<0.01	0.02	0.04	0.06	0.06	0.10	0.05

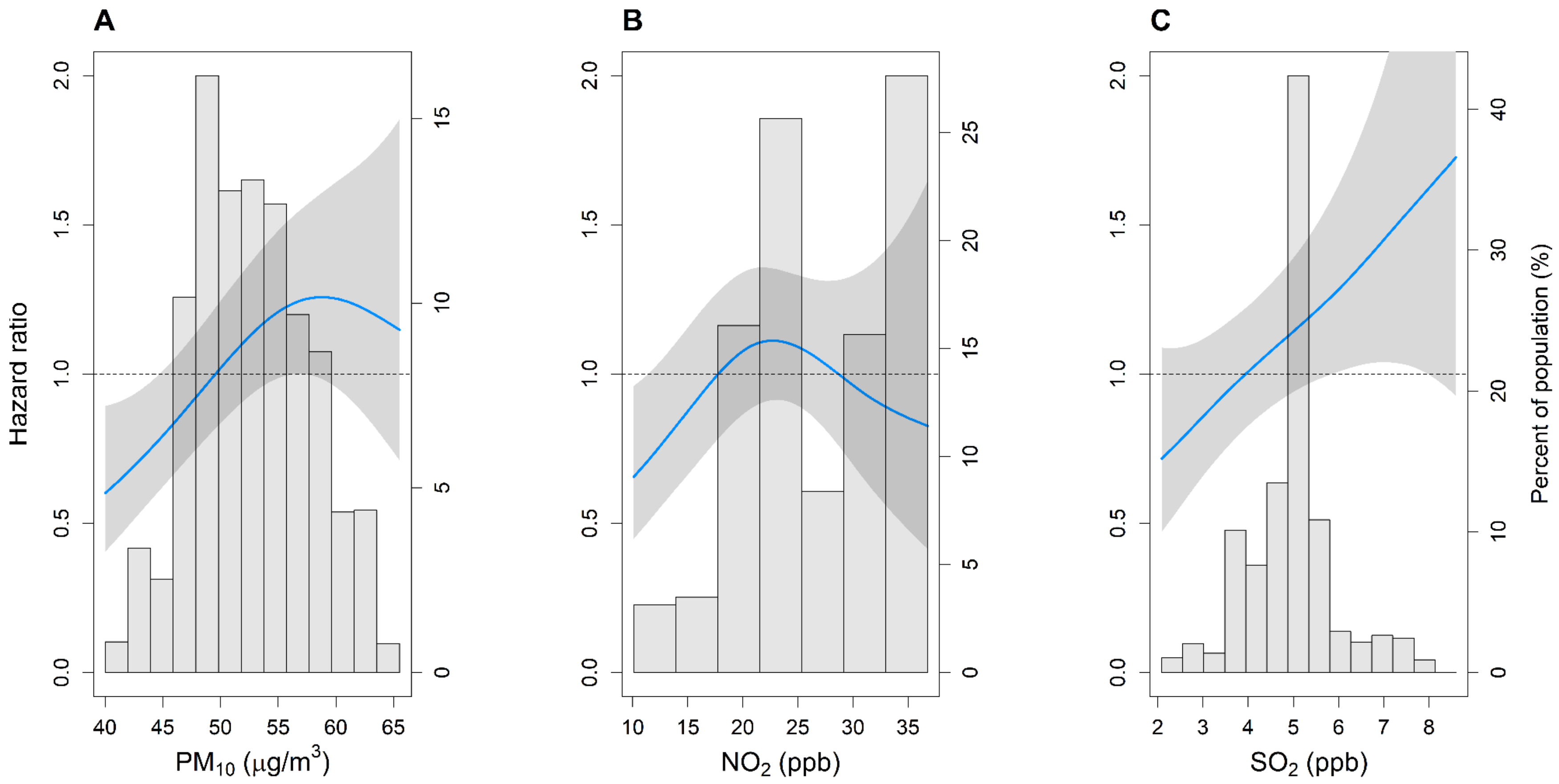

Figure S1. Concentration-mortality association (blue line) and 95% confidence interval (grey shadow) adjusted by natural spline with 2 degree of freedom in time-varying Cox model, with the percent distribution of population (histogram)

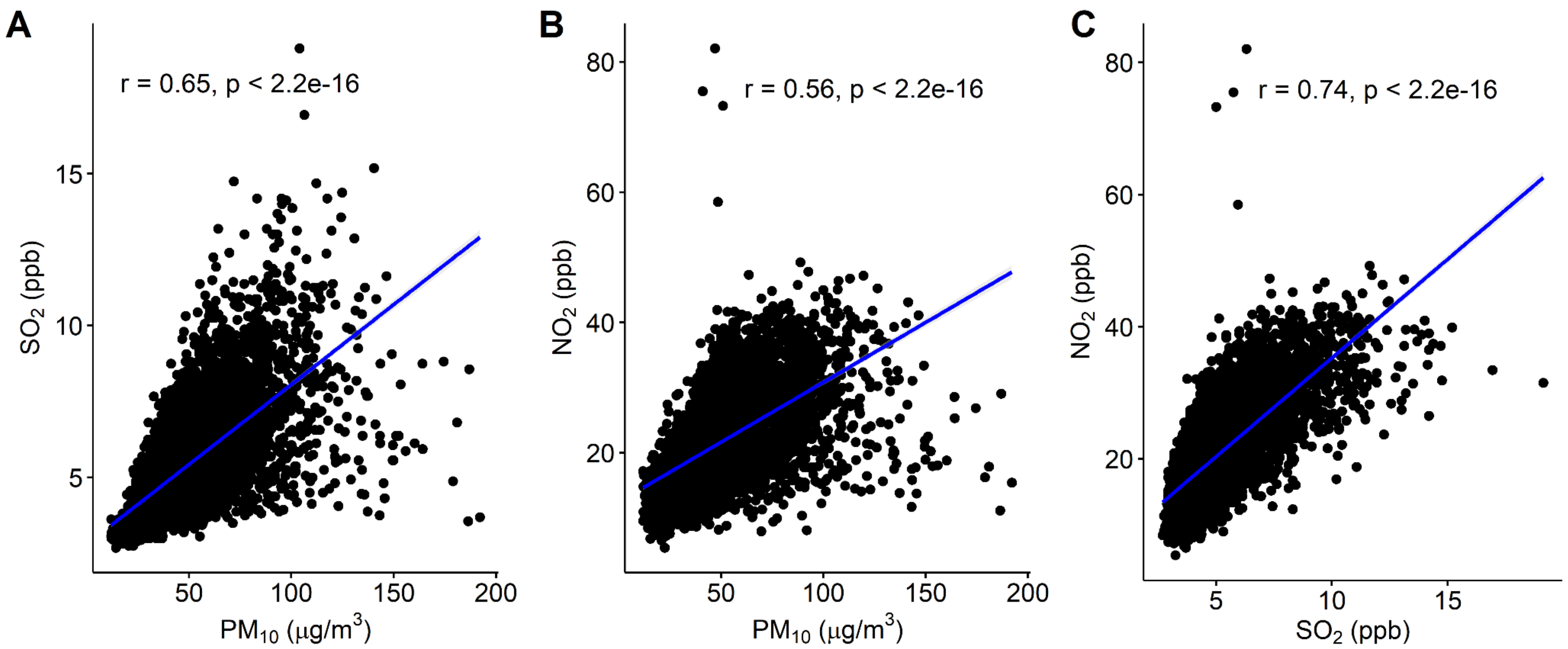

Figure S2. Correlation between daily concentrations of air pollutants between 2001 and 2015

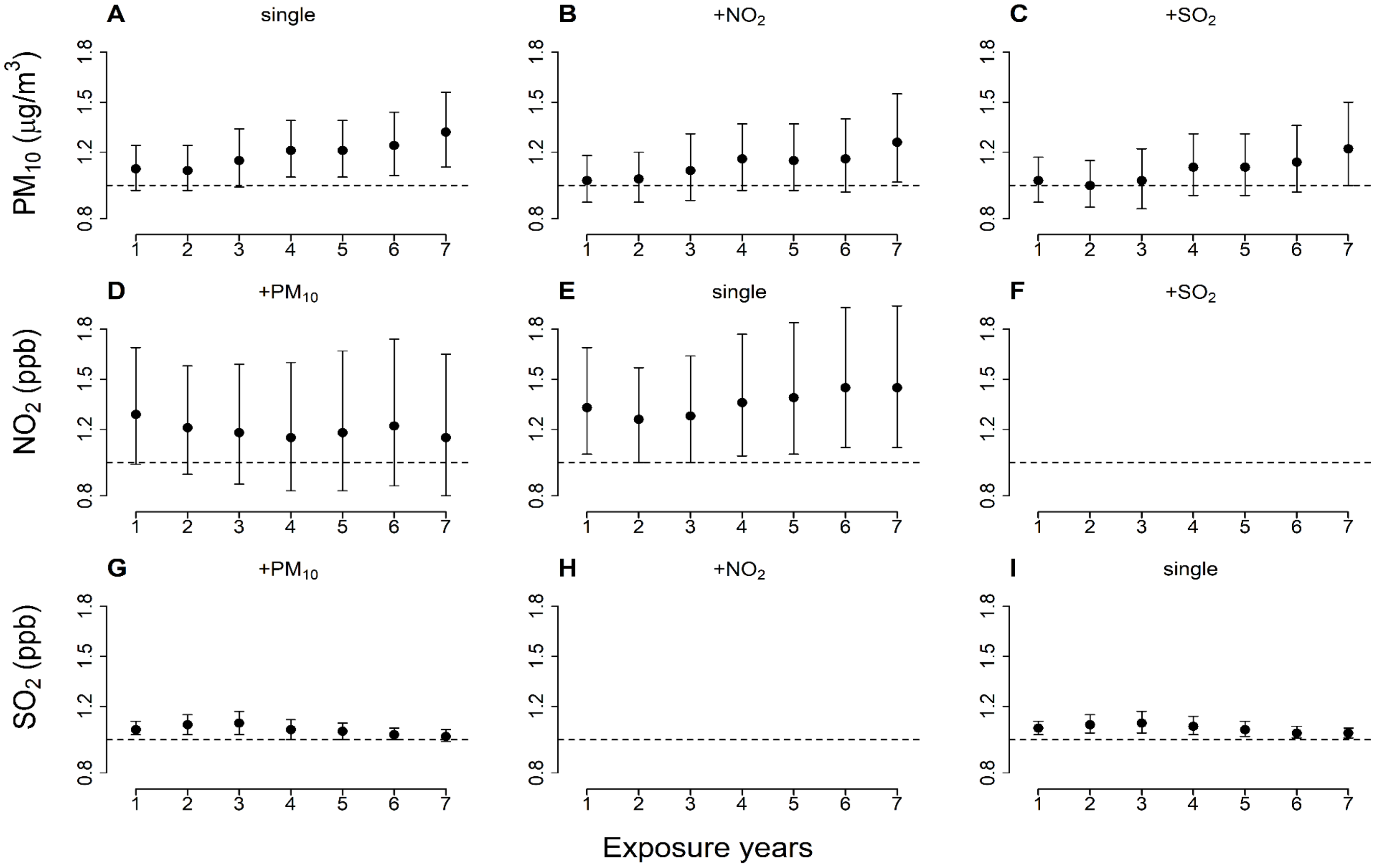

Figure S3. The association between mortality of ESRD patients and exposure to air pollutants in single- and two-pollutant models using time-varying (1yr-7yr) model
